# A Review of the Neural and Behavioral Consequences for Unitizing Emotional and Neutral Information

**DOI:** 10.3389/fnbeh.2013.00042

**Published:** 2013-05-27

**Authors:** Brendan D. Murray, Elizabeth A. Kensinger

**Affiliations:** ^1^Department of Psychology, Boston CollegeChestnut Hill, MA, USA

**Keywords:** emotion, associative memory, unitization, integration, medial temporal lobes

## Abstract

A special type of association, called a “unitization,” is formed when pieces of information are encoded as a single representation in memory (e.g., “shirt” and “blue” are encoded as a “blue shirt”; Graf and Schacter, 1989) and typically are later reactivated in memory as a single unit, allowing access to the features of multiple related stimuli at once (Bader et al., 2010; Diana et al., 2011). This review examines the neural processes supporting memory for unitizations and how the emotional content of the material may influence unitization. Although associative binding is typically reliant on hippocampal processes and supported by recollection, the first part of this review will present evidence to suggest that when two items are unitized into a single representation, memory for those bound items may be accomplished on the basis of familiarity and without reliance on the hippocampus. The second part of this review discusses how emotion may affect the processes that give rise to unitizations. Emotional information typically receives a mnemonic benefit over neutral information, but the literature is mixed on whether the presence of emotional information impedes or enhances the associative binding of neutral information (reviewed by Mather, 2007). It has been suggested that the way the emotional and neutral details are related together may be critical to whether the neutral details are enhanced or impeded (Mather, 2007; Mather and Sutherland, 2011). We focus on whether emotional arousal aids or inhibits the creation of a unitized representation, presenting preliminary data, and future directions to test empirically the effects of forming and retrieving emotional and neutral unitizations.

A key feature of human memory is the ability not only to remember discrete pieces of information but also to form novel associations between those pieces of information. Whether we are trying to identify the acquaintances who work at the same company or the specific combination of physical characteristics that indicate that a particular plant is poisonous, we frequently draw on memory for associations. A special type of association, called a “unitization,” can be formed when the pieces are encoded as a single representation in memory (e.g., the item “shirt” and the color “blue” are encoded as a “blue shirt”; Wollen et al., 1972; Graf and Schacter, 1989; Yonelinas, 2002; Diana et al., 2008, 2011). As Graf and Schacter (1989) describe, unitization can happen in one of two ways: either the items are perceived as having some underlying perceptual structure that leads them to be perceived as a single unit, or their co-occurrence implies a relationship that leads them to be combined together due to their presentation in space and time. Unitizations can be beneficial because they are typically reactivated in memory as a single unit, allowing access to the features of multiple related stimuli at once.

## The Creation and Retrieval of Unitizations

In this first section, we will review some of the neuroanatomy relevant to the formation and retrieval of unitized representations. We will also describe patient and neuroimaging data that can elucidate how unitizations are mnemonically encoded, and later accessed, in memory.

### Anatomical organization of the medial temporal lobes, and its relation to unitization

#### Hippocampus and surrounding cortices

The medial temporal lobes have been described as having a functionally hierarchical structure (Lavenex and Amaral, 2000; Montaldi and Mayes, 2010), consisting of the parahippocampal cortex (PHC), perirhinal (PrC), and entorhinal (EC) cortices, and the hippocampus proper. It is still debated whether these regions comprise a single system which enables declarative memory (e.g., Squire and Zola-Morgan, 1991; Squire et al., 2007) or contribute differentially to the encoding and retrieval of individual items and the relationships between those items (e.g., Cohen and Eichenbaum, 1993; Aggleton and Brown, 1999; Diana et al., 2007; Staresina and Davachi, 2008). Although coverage of this debate is outside the purview of this review, understanding of the proposed hierarchy is critical to discerning how unitized representations may be encoded and subsequently retrieved (Staresina and Davachi, 2006, 2008).

Briefly, the PrC and PHC have reciprocal connections with both unimodal (e.g., auditory and visual association areas) and multimodal association areas (e.g., posterior and anterior association areas; Lavenex and Amaral, 2000). PrC and PHC are believed to be the first site of integration of information from all sensory cortices, and data have suggested that there is functional specialization even at this first stage, with PrC primarily receiving object information from visual cortex and TE/TEO and PHC primarily receiving spatial information from the frontal and parietal cortices (Diana et al., 2007; Suzuki, 2010; also Bachevalier and Nemanic, 2008 for similar evidence from Rhesus macaques). As such, we can conceive of two possibilities for how the MTL may deal with unitized representations based on this differentiation. On the one hand, a to-be-unitized pair may be processed as a unitized object in regions within the dorsal or ventral visual processing stream (related to concepts of “object files” as originally conceived by Kahneman et al., 1992, and to the dual-system model of visual short-term memory proposed by Xu and Chun, 2006). This “pre-packaged” information could then be communicated to the PrC or PHC. On the other hand, the PrC may receive information about the separate to-be-unitized objects or object features and then begin the process of packaging them together – perceptually or mnemonically – into a single representation (e.g., Bussey et al., 2003; Bussey and Saksida, 2007; Kent and Brown, 2012) before passing on that information to the next regions in the hierarchy (see Cowell et al., 2010; Graham et al., 2010; Saksida and Bussey, 2010 for discussion of the role of the PRC in perception and memory for complex feature conjunctions).

Entorhinal cortex shares reciprocal connections with PrC and PHC, and it also receives direct inputs from multimodal association cortices. EC therefore receives input from all sensory modalities and is believed to play a greater role in the processing and integration of contextual information than of object information (Suzuki et al., 1997). EC does, however, receive object information from PrC and passes that information along to the hippocampus; most generally, the EC is considered to mediate much of the informational input to the hippocampus (Canto et al., 2008). Relative to other regions of MTL, less is known about the exact function of EC, but we can offer several possibilities. First, it is possible that because EC serves in this “gatekeeper” role of passing the hippocampus information from other brain regions (Hargreaves et al., 2005) – including PrC – that EC plays no direct role in the formation of unitizations. Instead, it may either pass pre-bound unitizations, formed in either the PrC or earlier regions of the ventral visual stream, to the hippocampus (if unitization happens before information reaches the hippocampus), or EC may pass information about individual objects or stimuli to the hippocampus, and unitization may take place within the hippocampus proper. Another alternative is that the EC itself is responsible for at least some processes relevant to forming unitizations, given its suggested role in contextual integration (Suzuki et al., 1997) and in subsequent correct recognition of verbal pairs (Jackson and Schacter, 2004). At present, these possibilities have not been distinguished, and so future research will be needed to explore the EC’s role in memory for unitizations.

The hippocampus sits atop the anatomical hierarchy and receives well-integrated information from EC, PrC, and PHC (Mayes et al., 2007). The hippocampus projects this richly complex, integrated information back to cortical association areas, and this process – the feedback loop of information from cortical association areas, through MTL, and back to the cortex – is believed by some to be what produces the phenomenology of episodic memory (Squire, 1992). As we have alluded to, although it is clear that the hippocampus is essential for the formation of typical associations among stimuli (Aggleton and Brown, 1999; Eichenbaum et al., 2007), it is less clear whether the hippocampus is needed for the formation and retrieval of unitizations (Giovanello et al., 2006; Quamme et al., 2007). We will return to this debate in the next section of this review.

It is important to note that the interactions between these MTL regions may be complicated by the fact that these regions can have functional influences on one another without having direct anatomical connections (see also Damoiseaux and Greicius, 2009 for discussion). Lacy and Stark (2012) revealed that functional connectivity – the strength of correlation in activity among brain regions – was stronger among cortical regions of the MTL (PrC, EC, and PHC) than it was between these regions and the hippocampus. Conversely, hippocampal subfields had strong inter-field functional connectivity but little connectivity with the surrounding cortex. As such, it may not be sufficient to explore what each of these MTL regions contributes to unitized memory in isolation; rather, understanding the functional connections between these regions will likely be critical to understanding the genesis of unitized representations in the brain.

#### Amygdala

In reviewing how emotion may interact with the unitization process, we must also consider the role of the amygdala. The amygdala’s role in the modulation of emotional memories – via its interaction with other MTL regions – has been well established (Gallagher and Chiba, 1996; Cahill and McGaugh, 1998; Kensinger and Schacter, 2006), and although its role has been investigated more thoroughly for memories that are fearful or aversive, it also plays a role in memories of pleasurable experiences (reviewed by LaBar, 2007). Animal research has demonstrated that the amygdala – particularly the lateral and basal nuclei – shares strong reciprocal connections with PrC, EC, and hippocampus, specifically in the subiculum and in subfield CA1 (Krettek and Price, 1977; Canteras and Swanson, 1992; Savander et al., 1997; Shi and Cassell, 1999; Pitkänen et al., 2000). Thus, there is substantial anatomical evidence indicating a reciprocal relationship between the amygdala and the anterior hippocampus as well as PrC. In addition, there is physiological and functional evidence of such a relationship. In rats, high frequency stimulation of the anterior hippocampus has been shown to produce long-term potentiation in the amygdala (Maren and Fanselow, 1985), and in patients, the amount of amygdala damage relates to the amount of hippocampal activity during an emotional memory task, and vice versa (Richardson et al., 2004). The amygdala also has robust connections to the visual cortex and regions along the ventral visual processing stream (Freese and Amaral, 2005; Duncan and Barrett, 2007). As such, there is an anatomical basis for predicting an interaction between the amygdala, PrC, and visual pathway, one that could influence the unitization process.

It has become clear that the amygdala does not act to enhance all aspects of a memory. Instead, the amygdala has selective effects on memory, enhancing some elements of a memory while having no beneficial impact – and sometimes even having an impairing effect – on other elements (reviewed by Kensinger, 2009; Mather and Sutherland, 2011). While not specifically discussing unitization, Mather (2007) described an “object-based framework” that is highly relevant to the process of unitization: when contextual details (such as color, temporal order, etc.) are viewed as being integral or intrinsic to the emotional stimulus, emotional arousal (likely via activation of the amygdala; see Kensinger et al., 2011 for evidence) enhances the binding of those details. This finding suggests that amygdala engagement can enhance the process of unitization.

Having reviewed the anatomical hierarchy within the medial temporal lobes, we now move to examine the empirical evidence for how these structures help produce episodic memory for items, their associations, and unitized representations. We begin with a brief discussion of a common way that memory for items and their associations is tested in the laboratory: through assessments of recognition memory. We focus on a model that argues that recognition memory can be supported by dissociable processes – recollection (including memory for episodic details) and familiarity (roughly akin to item familiarity; Atkinson and Juola, 1974; Yonelinas, 2002) – that have in turn been argued to map on specifically to the hippocampus and PrC, respectively (Aggleton and Brown, 1999; Diana et al., 2007; Bowles et al., 2010). Understanding how these regions and processes interact to produce an episodic memory is critical to understanding how the brain may encode, store, and retrieve unitized representations and how emotion may impact those processes.

### Memory through recognition

Often in the laboratory, memory for the relationship between two items is tested through recognition. In a typical paradigm, participants will study lists of unrelated paired associates (e.g., two semantically unrelated words, face-name pairs, etc.), and then memory for pairs or for individual items can be tested as part of an “old/new” recognition test. Although there are still some disagreements even among those who propose a dual-process model of recognition comprising recollection and familiarity (e.g., Yonelinas, 1994, 2002; Wixted, 2007), what is generally agreed upon is that recollection typically refers to the recall of specific episodic detail that was associated with the test stimulus: for example, a participant sees a recognition cue and retrieves specific knowledge unique to the context in which that cue was initially encountered, such as what information it appeared in conjunction with, what information temporally preceded or followed it, what thought was triggered by the information’s presentation, and so on (Montaldi and Mayes, 2010). Familiarity, on the other hand, is assumed to require no such access to episodic details. It is characterized by the knowledge that a particular test stimulus has been encountered previously – sometimes referred to as a “feeling of knowing” (Montaldi and Mayes, 2010) – but is not accompanied by retrieval of other contextual or otherwise associated details. Although there are alternative accounts to this “dual-process” model of memory (e.g., Squire et al., 2007), our goal in this review is not to adjudicate between the models. Rather, our review will follow from a wealth of research broadly demonstrating behavioral and neural dissociations between these processes.

### Unitization in the brain

A plethora of studies have supported a dual-process view not only of recognition memory but also of the MTL system (see Eichenbaum et al., 1994; Brown and Aggleton, 2001 for initial proposals). This research has revealed that the hippocampus is involved in associative binding of an item to its episodic context while the PrC plays a more dominant role in the representation of single items (see Diana et al., 2007; Eichenbaum et al., 2007 for reviews). An important caveat, however, and the one that we describe next, is that this hippocampal binding, and associated recollection, may not be necessary in cases of unitization.

Patient and imaging data support the notion that unitized representations can be supported by PrC and recognized via familiarity. To examine whether familiarity could contribute to the recognition of unitized representations, Diana et al. (2011) asked participants to view items presented on either a red or green background, and they were instructed either to imagine the item in the color of the background (the high-unitization condition), or to remember independently what color of background the item was paired with (low-unitization). Behaviorally, familiarity-based judgments were significantly higher in the high- vs. low-unitization condition, while recollection-based judgments did not differ. ERP data showed that the ERP correlates of familiarity – the early, fronto-central positivity – were significantly more apparent in the high- vs. low-unitization condition, while unitization demands had no effect on the ERP correlates for recollection. As noted by Diana and colleagues, their data – taken together with data from other studies that also revealed enhanced ERP correlates of familiarity on tasks encouraging unitization (e.g., Ecker et al., 2007; Diana et al., 2008; Bader et al., 2010) – provide relatively strong support for the proposal that unitization enhances familiarity-based recognition.

Although unitization may enhance the ability to recognize item associations on the basis of familiarity, Pilgrim et al. (2012) revealed that this may come at a cost when recognizing the individual items within the pair. They asked participants to study lists of single items, as well as pairs of words studied using either a unitization strategy (i.e., using mental imagery of the two items interacting in some way) or a non-unitization strategy (i.e., imagining the two items separately). Participants were then given a recognition test of single items from both the item-only and unitization/non-unitization conditions. At retrieval, the authors observed a reduced frontal effect, typically associated with familiarity-based retrieval, during recognition of items studied in the unitization condition relative to the item-only or non-unitization conditions. The authors interpret this result as suggesting that unitization limits familiarity-based access to items that have been combined into a unitized representation. Importantly, unitization did not affect overall item recognition accuracy: there was no difference in *d*′, hit rate, or reaction time for items in those two conditions. Thus, unitization affected *how* the information was recognized (by familiarity or recollection) and not *whether* it was recognized. Although the authors describe this reduced familiarity signal as a potential “cost” of unitization, they note that it could alternatively be interpreted as a benefit of unitization: equivalent item recognition memory, in an equivalent amount of time, is produced without reliance on a familiarity signal. Regardless of the exact interpretation of these findings, the results of this study, along with those of Diana et al. (2008), suggest that the process of unitization may differentially affect the role of familiarity in recognizing the associations compared to the individual items, enhancing the influence of familiarity on the former while reducing its influence on the latter.

Further evidence to suggest that unitization creates an associative representation that can be retrieved by processes typically associated with item memory or familiarity has come from two sets of data sampling patients with MTL amnesia. Giovanello et al. (2006) first showed, in a group of non-amnestic individuals, that recognition of unitized associations relies more on familiarity than recognition of non-unitized associations. They then discovered that patients with amnesia were better at differentiating between studied and rearranged pairs if the pairs formed compound words (e.g., correctly responding “old” to the studied words “blackmail” and “jailbird,” but “new” to the rearranged word “blackbird”) than if the pairs formed novel associations (e.g., “surgeon-arrow”). For non-amnestic controls, there was no such difference between the two types of associations. These data are consistent with the proposal that unitized representations can be remembered on the basis of item memory or familiarity, processes more likely to be spared in amnesia than those related to associative binding or recollection.

Quamme et al. (2007) presented similar findings: the researchers tested five amnesic patients, two of whom had undergone a left unilateral temporal lobectomy, damaging the hippocampus as well as PrC and EC, and three of whom were believed to have lesions restricted to the hippocampus as a result of cerebral hypoxia following cardiac arrest. When given pairs of unrelated nouns to study, hypoxic patients with intact rhinal cortices later recognized the pairs more readily if the words had been combined into a compound word with a novel definition (e.g., “cloud-lawn” would be defined as, “A yard used for sky-gazing.”) than if the words were presented in a sentence (e.g., “The ___ could be seen from the ___”). The two patients with temporal lobectomies, exhibiting damage to the rhinal cortices, did not show any such benefit from unitization.

These data offer compelling evidence that the hippocampus is not necessary for the encoding and retrieval of unitized representations, and they further suggest a role for the rhinal cortices in the formation of unitizations. Further evidence for a role of the PrC in unitization has come from studies using functional MRI. Using a paradigm similar to that of Quamme et al. (2007), Haskins et al. (2008) directly tested the hypothesis that PrC can support the encoding of unitized representations. Healthy participants were given pairs of semantically unrelated nouns (e.g., “steam tree”) and were asked to rate how well the words fit into a given sentence frame (no unitization), or they were given a novel definition for the compound of the two words and asked to rate how well they thought the definition fit the compound (unitization). They were then shown test pairs that were either intact from study or rearranged and had to indicate whether the words had been studied together or not. The imaging data, along with behavioral data from participants’ ROCs, indicated that left PrC was more active during compound than sentence trials, and that its activity at encoding was predictive of correct familiarity-based recognition judgments at test.

Staresina and Davachi (2006) also offer functional MRI evidence that the PrC can support the encoding of unitized item-color information. In their task, participants viewed the verbal label of an item (e.g., “elephant”) on a colored background, and were asked to imagine the item in the color of the background (e.g., for a red background, imagine a red elephant) and decide if the representation could plausibly appear in the real world or not. Participants were then given a surprise test in which they had to indicate if items were old or new, and if they judged an item to be old, they had to indicate with what color the item had been presented. While they observed that bilateral hippocampal activity at encoding predicted subsequent associative recognition success, encoding activity in left PrC was also found to be predictive of success at retrieving both the words and also the word-color pairs. They suggested that the PrC was able to support associative recognition for the item and its corresponding color because the color and item information had been unitized at encoding.

To directly test their hypothesis, in a second study, Staresina and Davachi (2008) used a similar encoding task, but they also asked the participants to make one of two semantic decisions about the item (judging its pleasantness or its plausibility). This manipulation allowed the researchers to assess how MTL subregions contribute to memory for those different types of detail. Consistent with their previous results, they found that encoding activity in the left hippocampus and bilateral PrC predicted subsequent associative recognition for item-color pairings. In contrast, only the hippocampus and not PrC predicted successful retrieval of the item-context (i.e., encoding task) association. If we consider the color to be associated via a process of unitization but the encoding task to be associated through other means, then these data offered further evidence that PrC may indeed help to encode unitized associations but not other forms of associative learning.

While these three functional MRI studies indicate that PrC offers an important contribution to the formation of associative memory through unitization, they still cannot elucidate the specific role that PrC might play in the unitization process. That is, it still remains unclear from these data whether PrC is responsible for assembling unitizations – actually concatenating together the different object features into a single unit – or if it may receive already-unitized representations from other regions and PrC encodes that pre-assembled representation. To test this, Staresina and Davachi (2010) conducted a third study systematically varying the unitization demands at encoding to investigate how PrC would track with increasing unitization requirement. The hypothesis was that if PrC activity showed a variable relation based on the varying unitization demand, it would suggest that the PrC plays a role in actually putting together novel unitizations. In their study, participants viewed objects that were intact, fragmented into two parts, or fragmented into four parts, and participants were asked to imagine the whole item in the color of the background and to determine its real-world plausibility. By including three fragmentation conditions, the researchers were able to vary how much the item needed to be unitized to succeed at the encoding task. Participants were given the same recognition task from Staresina and Davachi (2006), described above. As in Staresina and Davachi (2006), PrC activation at encoding – this time, bilaterally – was predictive of subsequent item and associative recognition success; however, PrC activation was unaffected by level of fragmentation. Rather, regions throughout the ventral visual stream tracked with increasing unitization demand. These data suggest that while PrC can support the mnemonic encoding of unitized associations, the perceptual integration of a unitized representation may occur in earlier stages of processing.

The role of the PrC in unitization likely does not stop after encoding. Diana et al. (2010) asked participants to study nouns presented on a red or green background and to process the color either as an item feature – by imagining the item in that color – or as a contextual detail by imaging the item interacting with a stop sign (red) or dollar bill (green). Although control participants were able to retrieve the color information with equal probability, regardless of the encoding instructions, amnesic patients were more likely to retrieve the color information if it had been processed as an item feature rather than a contextual detail. fMRI results with healthy individuals revealed that PrC activity was associated with the retrieval of the color information if it had been studied as an item feature while PHC and hippocampal activity was associated with the retrieval of the color information if it had been studied as a contextual feature.

It is worth noting that domain in which information is presented and manipulated (e.g., whether the participant is creating a compound word or imagining the visual representation of an item) likely affects the neural processes that underlie successful unitization. Although to our knowledge, no study has directly contrasted the neural processes leading to integration by visual or verbal means, it seems plausible that tasks that require unitization of visual entities – such as those just described – would engage visual processing regions while tasks that achieve unitization through the creation of a new concept may engage semantic processing regions such as anterior temporal lobe (Lambon et al., 2010). Regardless of the domain in which the unitization occurs, however, one critical point remains true: it does not appear to be the case that hippocampal engagement is necessary for unitization to be successful.

The data reviewed in this section suggests three main conclusions: (1) Unitized associations can be remembered on the basis of familiarity and using processes typically associated with single item memory, but this effect of unitization may come at the cost of familiarity-based recognition of the individual components of the associated pair. (2) At an anatomical level, the data suggest that the hippocampus is not necessary for forming and encoding unitizations. Instead, PrC may play a role in the mnemonic encoding and retrieval of those unitizations, although (3) at encoding, PrC may, instead or in addition, receive already-unitized information from other brain regions and then work to store that unitized representation into long-term memory. In the section below, we outline the predictions that each of these findings makes with regard to the effect of emotion on memory for unitized representations.

## Emotion and Unitization

It has been well established that emotional information is handled differently in memory from non-emotional information, with emotional information typically receiving a mnemonic benefit (reviewed by Hamann, 2001; Kensinger, 2009). The emotionality of an experience is often characterized along two orthogonal dimensions: arousal (how exciting or calming an experience is) and valence (how pleasant or unpleasant; Russell, 1980). Because the bulk of research examining the effect of emotion on memory has focused on the dimension of arousal (Cahill et al., 1994; Cahill and McGaugh, 1995), and because arousal is thought to be the main factor that influences amygdala activity (Adolphs et al., 2001; Sharot and Phelps, 2004; Berntson et al., 2007), we will focus our review on the contributions of emotional arousal to emotional memory. It is important to note, however, that valence can influence associative memory as well (Pierce and Kensinger, 2011) and may interact with arousal to influence both the subjective qualities of a memory (Talarico and Rubin, 2003; Sharot and Phelps, 2004; Zimmerman and Kelley, 2010) and also the way that the amygdala interacts with visual and prefrontal processes (e.g., enhanced connectivity between amygdala and middle occipital gyrus and amygdala and inferior frontal gyrus during encoding of high-arousal negative items and low-arousal positive items; Mickley Steinmetz et al., 2010).

To date, there has been little research that specifically has examined the effect of arousal on unitization. There have, however, been many studies that have more generally assessed the effect of arousal on recollection and familiarity, and on the associative binding of item features and contextual details. In the sections below, we review the research on the effects of arousal on memory that we believe can lead to informed predictions regarding the effects of arousal on unitization.

### The effects of arousal on associative binding: Implications for unitization

Although the effects of arousal on associative binding initially seemed inconsistent – with arousal sometimes enhancing the binding of details (e.g., MacKay et al., 2004) and at other times having no effect, or impairing, such binding (e.g., Kensinger and Schacter, 2006; Bergmann et al., 2012; note that the latter examines interactions between both valence and arousal) – more recent accounts have proposed a unified framework for understanding the complex pattern. Mather (2007) proposed an “object-based” framework, with arousal enhancing the binding of information encoded as an item feature but not of information encoded as a contextual detail (see also Kensinger, 2007, 2009). This framework can account for much of the extant data, but there are some exceptions, when arousal does not enhance memory for intra-item features (Guillet and Arndt, 2009) and when it does enhance memory for inter-item binding (Pierce and Kensinger, 2011).

Recognizing the need to account for these contradictory patterns, Mather and Sutherland (2011) proposed the “Arousal-Biased Competition” (ABC) model, describing how arousal may bias resources toward the most conspicuous or goal-relevant stimuli. Thus, if a single item (e.g., a snake) gains priority, then arousal will enhance the binding of the features of that item (e.g., its color, form). But if the pairing of items takes on importance (e.g., the snake and its owner) then arousal will enhance the binding of that association. In other words, it is not the *type* of detail (item vs. contextual or intra- vs. inter-item associations) that predicts the effect of arousal but the *goal relevance* of the detail (see related discussion by Levine and Edelstein, 2009). Although it is always the case that goal-relevant information will be prioritized, according to ABC, arousal exaggerates this prioritization; thus, arousal will enhance the binding of goal-relevant features and will impair the binding of features that are not goal relevant.

In this context, the effect of arousal on associative binding will depend critically on the way the to-be-bound pieces of information are initially processed or perceived. On the one hand, because unitization requires that pieces of information be processed as a coherent whole, all features that are being bound into a unitized representation may be goal relevant. If true, then arousal may lead to a beneficial effect on the process of unitization. On the other hand, because arousing features can capture attention and become prioritized for processing, the presence of those arousing features may make it harder to attend to the other features present at the same time, thereby making the creation of a unitization harder to achieve. Each of these possibilities will be expanded upon in the next sections.

### Why arousal may enhance unitization

As described earlier, neuroimaging data (e.g., Staresina and Davachi, 2010) suggest that visual regions such as those within the ventral visual pathway can be responsible for the initial formation of unitized representations. Thus, unitizations may be created before the information reaches the MTL system. Separate literatures have revealed that activity in these visual regions can be modulated by the arousing content of information. Neuroimaging studies have shown that participants tend to exhibit greater activity in visual cortex and ventral visual stream when viewing emotional vs. non-emotional images (Vuilleumier et al., 2001, 2004; Compton, 2003; Mather et al., 2006), likely because of modulation of visual processing by the amygdala. These data suggest that processing of emotional items within the visual cortex may be prioritized, leading these items to be attended to before neutral items (Öhman and Mineka, 2001; Öhman et al., 2001), to hold attention longer (LaBar et al., 2000; Nummenmaa et al., 2006; Knight et al., 2007), and to be associated with greater encoding activity in visual regions than non-emotional items (Bradley et al., 2003; Mather et al., 2006).

Putting these literatures together: if visual regions such as those along the ventral visual pathway are responsible for the initial formation of unitized representations, the presence of arousal could facilitate activation within those regions, enabling the rapid creation of a unitization. In addition, these literatures may suggest that unitizations containing an arousing component would require less cognitive effort to create than unitizations that contain only neutral information. Because arousing information benefits from prioritized processing, it may be more rapidly and easily integrated unitized. On the other hand, neutral information – which is not privy to such prioritization – may require more cognitive effort to successfully unitize.

Several investigations have demonstrated that when non-emotional information is encoded as a feature of an emotional item, memory for the relationship between the emotional and non-emotional information is enhanced. For example, two recent studies (Mather and Nesmith, 2008; Nashiro and Mather, 2010) demonstrated that when participants passively viewed non-emotional and emotionally arousing pictures at different locations on a computer screen, their memory for the location of emotionally arousing pictures was significantly better than their memory for the location of non-emotional pictures. Mather and Nesmith (2008), in particular, offer evidence for the above points about emotional information being integrated with relatively little cognitive effort: In Experiment 4 of that investigation, the authors varied the amount of time participants had to view pictures (relative to the amount of study time provided in the first three experiments) to determine if location memory tracked with the amount of time participants had to view pictures. The authors found that memory for the location of pictures was independent of how long the pictures were presented. Moreover, it was shown that picture-location conjunction memory for non-emotional pictures was not impaired when those pictures were presented at the same time as emotional pictures, even when encoding time was limited. From this evidence, the authors conclude that arousing pictures did not capture attention for any extended period of time (or else picture-location memory for concurrent non-emotional pictures would be impaired), suggesting that picture-location binding for arousing pictures must happen quickly and with relatively little effort.

Another recent behavioral study (Guillet and Arndt, 2009) demonstrated that inter-item memory could be enhanced by arousal, as well. Mnemonic binding of verbal pairs was enhanced if one of the target words was arousing (i.e., a “taboo” word). Memory for the association between the taboo word and a neutral word was enhanced relative to when the neutral word was paired with another neutral word, or paired with a negative non-arousing word. Though these data offer evidence that arousal may enhance the formation of inter-item associations, it is important to note that relatively little is known about what makes taboo words – and their associations – memorable. Madan et al. (2012), for example, demonstrated that arousal is only one dimension that separates taboo words from other emotional high-arousal and emotional low-arousal words. Those authors used multidimensional scaling for several dimensions of normed ratings (e.g., “familiarity,” “offensiveness,” “imageability”) of neutral, emotional, and taboo words to show that arousal alone does not clearly differentiate taboo words from other emotional words; rather, some undetermined combination of other stimulus characteristics is likely to better differentiate those stimuli. Therefore, it is unclear whether it is the arousing nature of taboo words that leads them to be better associated in Guillet and Arndt (2009), or if some other set of characteristics – like offensiveness or “tabooness” that may be separate from arousal – is driving those effects. Moreover, Madan et al. (2012) demonstrate that across a number of pair types – with pairs containing only taboo, only moderately arousing negative, only neutral, or any combination thereof – the presence of emotional arousal only ever improved item memory, and never improved association memory.

### Why arousal may impair unitization

The prior discussion of goal relevance highlights an important caveat regarding the beneficial effect of arousal on unitization: Participants would need to find all of the to-be-unitized components to be goal relevant in order for arousal to facilitate their unitization. This goal relevance would likely be achieved in a task that directed participants to integrate the various features, as the task instructions would make both items relevant. But if integration is not emphasized, arousal may instead impair the unitization by focusing perceptual and cognitive resources only on the arousing feature, thereby preventing unitization with the other features. If attention is directed to the arousing information and not distributed to all elements of the to-be-unitized set, the unitization may fail.

Arousing information can capture visual attention and can be processed in a prioritized fashion. Studies that record participants’ eye gaze patterns while they view either arrays of emotional and non-emotional stimuli or complex visual scenes have shown that participants initially saccade to emotional information more quickly, and they saccade more frequently to the emotional information than to neutral information (LaBar et al., 2000; Öhman and Mineka, 2001; Öhman et al., 2001; Mather and Knight, 2006; Knight et al., 2007). In studies that require participants to detect two or more target items in a rapidly presented stream of stimulus items, a phenomenon known as the “attentional blink” is typically observed: participants readily detect the first target item in the rapid stream, but often miss the subsequent target item (e.g., Chun and Potter, 1995). The presumption is that attentional resources are directed toward detecting the first target item, resulting in the second item being missed. However, when the second target item is emotionally arousing, the attentional blink effect is attenuated. Despite presumably depleted attentional resources, participants can still detect the emotional second target item (Anderson and Phelps, 2001; Keil and Ihssen, 2004; Anderson, 2005). These findings suggest that when attentional resources are limited, emotionally arousing items are preferentially processed over neutral items.

This prioritized processing of the arousing information can result in poor processing of temporally- or spatially-proximate information (e.g., Easterbrook, 1959). These factors can combine to create instances in which the arousing elements of an event (e.g., a smashed car) are remembered at the expense of the surrounding, non-emotional details (e.g., details of the street on which the accident occurred; see reviews by Buchanan and Adolphs, 2002; Reisberg and Heuer, 2004; Levine and Edelstein, 2009). Indeed, in laboratory studies using complex visual scenes as encoding stimuli, a “trade-off” in memory is often observed: participants exhibit better memory for emotional than non-emotional items that appeared in the scenes, and their memory for the background details is better when those backgrounds are presented with neutral rather than emotional items (e.g., Kensinger et al., 2005). Neurally this trade-off is associated with increased activity in the middle temporal gyrus, among other regions (Waring and Kensinger, 2011). This region within the ventral visual stream is more active during the encoding of scenes for which the emotional item is subsequently remembered and the background forgotten than for scenes in which both the item and background are remembered, suggesting that visual resources may be preferentially processing the emotional information at the expense of the non-emotional information.

These findings may relate to the process of unitization for an emotional and a non-emotional feature. If sensory-processing regions preferentially process the emotional features over the non-emotional feature, the success of the unitization could be compromised. By giving preference to the emotional information, the unitization may not equitably represent both the emotional and non-emotional components. Instead, the unitization may be dominated by the emotional information, or the unitization may fail altogether because processing resources are focused on the emotional information to the exclusion of the non-emotional details.

Further evidence that emotion may impair the formation of unitizations – particularly in the case of negative emotional information – comes from a study by Okada et al. (2011). In that study, participants were asked to learn novel pairs consisting of neutral faces paired with positive, negative, or neutral words while undergoing an fMRI scan. The researchers found a strong negative correlation between left amygdala activity during the encoding of negative pairs and subsequent memory performance, suggesting that amygdala activation at encoding may disrupt the formation of negative associations. It is important to note, though, that the opposite effect was observed for positive face-name pairs: increases in amygdala activation were related to better subsequent memory for those pairs, suggesting that the effect of arousal may depend on the valence of the information to be remembered.

### The mnemonic influences of arousal: implications for unitization

One of the most-often replicated effects of arousal on memory is the creation of a subjectively vivid memory (Phelps and Sharot, 2008; Sharot and Yonelinas, 2008; Zimmerman and Kelley, 2010). The beneficial effects of arousal on memory are often detected on tasks that assess not only recognition rates but also the subjective qualities of a memory. For instance, even when recognition rates do not differ between arousing and neutral stimuli, participants are often still more likely to say that they vividly recollect emotional items (Ochsner, 2000; Talarico and Rubin, 2003; Sharot et al., 2004). Although this effect may sometimes reflect a biased endorsement of emotional memories (Windmann et al., 2002; Dougal and Rotello, 2007), sometimes it seems to correspond with an improved ability to remember at least some features of an arousing item’s presentation (Neisser and Harsch, 1992; Kensinger and Corkin, 2003; see review by Mather, 2007). By contrast, arousal often does not enhance the familiarity of items, and when it does, it often seems to do so for both studied and non-studied items alike, increasing both correct and incorrect endorsements and having little effect on memory discriminability (e.g., Sharot et al., 2004).

Consider that one of the predominant reasons why there is a mnemonic benefit for unitizations may be because of their ability to be recognized on the basis of familiarity (Diana et al., 2008, 2011; but see Mickes et al., 2010). If so, then the literature just reviewed may suggest that the relative benefit of unitization would be less for emotional associations than for neutral associations. That is, arousal may enhance processes that are connected to the recollection of information more than those connected to the familiarity of information, while unitizations may do the opposite.

At a neural level, it is less clear whether there is a distinction between the memory regions most influenced by arousal and those implicated in unitization. Certainly, much emphasis has been placed on the ability for arousal to modulate *hippocampal* function, via connections between the amygdala and the hippocampus. The initial studies examining the effect of arousal on memory consolidation focused on interactions between the amygdala and hippocampus proper (reviewed by McGaugh, 2004) and subsequent neuroimaging studies in humans have followed suit (reviewed by Phelps, 2004), often focusing on the correlations or co-activations between the amygdala and the hippocampus during the formation or retrieval of an emotional memory (e.g., Dolcos et al., 2004; Kensinger and Corkin, 2004). Yet the amygdala also shares strong reciprocal connections with the PrC (Pitkänen et al., 2000; Kajiwara et al., 2003). A recent study revealed that stimulation of the amygdala could lead to induction of synaptic plasticity (i.e., long-term potentiation) within the PrC (Perugini et al., 2012), demonstrating a functional connection between the two regions. Thus, the finding that unitizations rely less on the hippocampus and more on the PrC makes no strong prediction with regard to the effect of emotion on unitization. The reciprocal connections between the amygdala and the PrC appear to be sufficiently strong to enable arousal-driven modulation, though of course it remains an open question whether such modulation would occur during the encoding or retrieval of unitizations.

As noted earlier, if unitizations rely on processes within the ventral visual processing stream, the amygdala activation that occurs during the experience of arousal would be likely to modulate processing within that stream (Anderson, 2005; Vuilleumier, 2005). There is ample evidence that the amygdala sends strong back-projections to regions through the sensory cortices (Amaral and Price, 1984; Iwai and Yukie, 1987; Morris et al., 1998), and this prioritized processing may facilitate the creation of a unitization. Thus, arousal could enhance the process of unitization either through modulation of medial temporal-lobe processes or through modulation of earlier sensory processes.

### The effects of arousal on unitization: preliminary evidence

We Murray and Kensinger (2012) conducted a study to examine the effect of arousal on associative binding when unitization is encouraged. We asked participants to study pairs of words and to either maintain separate mental images of the items (non-integrated) or to create an integrated, interactive mental image of the two items (integrated), a task similar to that described in Pilgrim et al. (2012). Some pairs contained an emotional and a neutral word, and other pairs contained two neutral words. Each study pair was displayed for 2, 4, or 6 s, and after each pair participants were asked to report the vividness of the two individual images (non-integrated) or one unified image (integrated) they were able to generate.

Participants consistently rated their imagery success as being high for emotional pairs, regardless of the length of the encoding trial, but they reported little success in generating images for neutral pairs during short (2-s) encoding trials. These results are consistent with the prediction that arousal would facilitate the process of unitization: When arousal was present, participants were able to create an image that integrated two distinct items, even with little time to do so.

Participants were then given a surprise cued recall test in which they were given a single studied word and asked to provide its paired counterpart. Because this was an associative task, we unsurprisingly found that pairs studied in the integration condition were better recalled than those studied in the non-integration condition. Conversely, on an item recognition test, performance was better in the non-integrated condition than in the integrated one. This finding is generally consistent with evidence presented earlier, suggesting that although the formation of unitizations can enhance memory for associations, this unitization sometimes comes at the cost of gaining access to the individual item representations (Pilgrim et al., 2012).

The twist, however, was that the integration benefit was disproportionately *greater* for neutral pairs than for pairs containing an emotion word. Although participants had an easier time generating the integrated images for the emotional pairs, they showed *less* of a mnemonic benefit from integration of those pairs. At the broadest level, these data suggest that *forming* unitizations – actually assembling together the different perceptual and semantic features – and *remembering* those unitizations in long-term memory could be differentially affected by arousal. It is interesting to note that this distinction parallels one noted by Zimmerman and Kelley (2010); they reported that participants gave higher judgments-of-learning to emotional pairs than to neutral ones but actually remembered the emotional pairs less well. When reviewing this study, Madan et al. (2012) suggested that the participants may have applied less effort when forming associations of those emotional items, an explanation that is also consistent with our data. It may be the case that if information is more easily unitized – perhaps via processes implemented in visual processing regions – the mnemonic storage of that unitization is less effortful and therefore less durable.

## Future Directions and Conclusion

### Future directions

The research we have reviewed suggests the importance of distinguishing the effects of arousal on the initial process of forming unitizations from the process of storing those unitizations over the long term. Future research would do well to examine whether – if these processes were distinguished, perhaps using methods similar to Staresina and Davachi (2010) – arousal would have different effects on these two general types of processes.

It is also possible that the presence of arousal may lead to a shift from conceptual to perceptual binding processes in creating unitizations (see Graf and Schacter, 1989 for discussion of the contribution of these different processes). In discussing the neural architecture that may underlie encoding of emotional and non-emotional unitization, we have offered that emotional unitizations may be supported by interactions between the amygdala, ventral visual stream, and PrC, suggesting that such unitizations may rely on more perceptual linkages (e.g., what a poisonous plant looks like). Non-emotional unitizations, by contrast, may more often be supported by conceptual processes, perhaps requiring stimulus elaboration to effectively concatenate (e.g., realizing that the blanket, watermelon, and basket go together as a picnic). Although further empirical testing of this hypothesis is required, a first set of neuroimaging data (Murray and Kensinger, 2013) is consistent with this hypothesis, with successful integration of emotional pairs resulting in disproportionate activation within visual regions and successful integration of neutral pairs resulting in greater activation within prefrontal regions. Moreover, amygdala activity during negative integration was negatively correlated with activity in prefrontal and MTL regions, suggesting that amygdala engagement may disrupt prefrontal and MTL processes, consistent with the finding of Okada et al. (2011).

Emotional arousal, then, may shift unitization processes away from more conceptual ones implemented by prefrontal engagement and toward more perceptual ones implemented within visual regions. These different types of processes (perceptual vs. conceptual) may have differential downstream effects on subsequent memory for the unitizations. Conceptual elaboration may be a slower, more laborious process, but it may lead to a more deeply encoded, and more durable, representation. By contrast, perceptual integrations may happen rapidly, but be less durable over time. In other words, there may be a levels-of-processing effect (Craik and Lockhart, 1972), leading to a stronger and more durable memory trace for unitizations formed using prefrontal and MTL processes than for unitizations formed with disproportionate use of visual processes.

It would be informative for future research to examine how arousal affects the durability of unitizations. On the one hand, if the main effect of arousal relates to the way in which unitizations are initially formed, and if the processes creating unitizations for arousing items are less effortful and less “deep,” then it might be expected that the decay rate for emotional unitizations would be faster than for neutral unitizations. On the other hand, however, the consolidation hypothesis (Müller and Pilzecker, 1900), suggests that after information is initially encoded it remains in a fragile state before being solidified into memory (see review by McGaugh, 2000). The consolidation process is relatively slow, and one proposed reason is to give any associated emotional response sufficient time to influence the consolidation process (McGaugh, 2000; Phelps, 2004). Indeed, there is robust evidence that arousal often enhances the consolidation of information, such that the beneficial effects of arousal are more likely to be apparent after longer delays than after shorter ones. This pattern of results has been shown not only in assessments of item memory (Sharot and Phelps, 2004) but also in tests of associative memory (Pierce and Kensinger, 2011; however, see Szpunar et al., 2012 for evidence that memory for details of imagined future autobiographical events is better for positive and negative than neutral events after a 10-min delay, but better for positive and *neutral* than negative events after a 24-h delay). Thus, it is possible that even if emotional unitizations are less “deep” and are maintained less well over relatively short delays (e.g., 30 min), they may have a shallower forgetting curve than neutral unitizations, making them more durable over longer delays. Adjudicating between these alternatives – by assessing memory for unitizations after multiple delays, or by disrupting consolidation through a retroactive interference task – would appear to be important for determining whether arousal primarily exerts its effects on unitization through processes that occur as the unitization is initially formed or whether the effects continue as that unitization is stored.

As we have noted earlier, the neural processes that underlie the formation and retrieval of unitizations are likely to depend in some way on the verbal or visual demands of the unitization. Although verbal unitizations (e.g., novel compound words; Giovanello et al., 2006; Quamme et al., 2007; Haskins et al., 2008) and visual unitizations (e.g., assembling a fragmented item and imagining it in a specified color; Staresina and Davachi, 2010) share some similarity – that is, they can be performed without reliance on hippocampal processing – no study has directly contrasted the neural correlates supporting their creation or retrieval. Emotion may also affect the unitization process differently, depending on the modality of processing. The amygdala can influence both visual processing (Vuilleumier et al., 2001, 2004; Compton, 2003; Mather et al., 2006) and semantic processing (Skipper et al., 2011), but the extent to which those influences differ for visual and verbal unitization – or on tasks that require some combination thereof (e.g., Staresina and Davachi, 2006, 2008; Murray and Kensinger, 2012) – is currently unknown. Therefore, we believe a fruitful future investigation would employ both verbal and visual unitization of emotional and non-emotional information, in an attempt to elucidate the similarities and differences between those two domains.

One area that we have not discussed to this point is how unitization processes may be utilized in the creation of autobiographical memories. Current theories of autobiographical retrieval suggest that it is a reconstructive process (see reviews by Conway, 1996; Holland and Kensinger, 2010) in which memories of our past are reconstructed at retrieval from our stores of autobiographical knowledge. This reconstructive nature imparts flexibility on retrieval from events in our past: the same event may be described differently at different time points. Autobiographical details also can be intentionally recombined into past events that did not occur, or into simulated, plausible future events. In one fMRI study, Addis et al. (2009) asked participants to report details of autobiographical events (i.e., the “who,” “what,” and “where”) during a pre-scan session, and then directed those participants to imagine novel past and future events that consisted of recombined details from participants’ actual memories. For example, if the participant reported memories of “Jess buying a lottery ticket at a convenience store,” “Eating chicken fajitas with Cathy at Border’s Café,” and “Buying a flat screen TV with John at Best Buy” the participant may be asked to imagine a novel past or future event that involved “John,” “Border’s Café,” and “lottery ticket.” One could conceive of this process as being similar to unitization in that participants must take previously unrelated details and combine them into a single representation. A key difference however, is that in these tasks participants are asked to generate a representation of a new *event*, localized in time and space. To our knowledge, no tasks of unitization require this type of temporal or spatial localization of the representation. Perhaps because of this key difference, in the studies by Addis et al. (2009), this imaginative generation was shown to recruit activity in bilateral hippocampus, as well as activity in anterior prefrontal cortex and angular gyrus. These results suggest that whereas unitization of two words or items into a new representation may be accomplished in the absence of hippocampal processing (e.g., Giovanello et al., 2006; Staresina and Davachi, 2010), when new representations are created as *events* anchored in time and space, this may require the hippocampus.

### Conclusions

The literature reviewed here suggests that unitized associations can be formed and retrieved using dissociable processes from those used to support memory for other associative memories. Unitized associations can be remembered on the basis of familiarity and in the absence of hippocampal function. Their formation appears to be supported by some combination of processing within sensory-processing regions and within the rhinal cortex, although additional research is needed to further specify the involvement of these regions.

At least within a laboratory setting, when unitization is the specified goal, arousal appears to enhance the ability to form an integrated representation; this representation can be formed with high success even when under time pressure. Yet arousal does not enhance the ability to remember that unitized representation over the long-term, revealing a distinction between facilitated *formation* of a unitization and facilitated *retention* of that unitization. Further research will be needed to elucidate the reasons for these distinctions, but we suggest that arousal may facilitate the formation of unitizations via perceptual processing, whereas unitization for neutral items may be more likely to recruit conceptual processes implemented within prefrontal and MTL regions.

## Conflict of Interest Statement

The authors declare that the research was conducted in the absence of any commercial or financial relationships that could be construed as a potential conflict of interest.
